# Infant Neutropenia Associated with Breastfeeding During Maternal Antiretroviral Treatment for Prevention of Mother-to-Child Transmission of HIV

**DOI:** 10.4137/RRt.s13267

**Published:** 2014-02-13

**Authors:** Irene Njuguna, Marie Reilly, Walter Jaoko, Christine Gichuhi, Gwen Ambler, Elizabeth Maleche-Obimbo, Barbara Lohman-Payne, Tomáš Hanke, Grace John-Stewart

**Affiliations:** 1 Department of Pediatrics and Child Health, University of Nairobi, Nairobi, Kenya.; 2 Department of Medical Epidemiology and Biostatistics, Karolinska Institute, Stockholm, Sweden.; 3 Kenya AIDS Vaccine Initiative (KAVI), Department of Medical Microbiology, University of Nairobi, Nairobi, Kenya.; 4 Department of Clinical Medicine and Therapeutics, University of Nairobi, Nairobi, Kenya.; 5 Department of Global Health, University of Washington, Seattle, WA, USA.; 6 The Jenner Institute, University of Oxford, Oxford, United Kingdom.; 7 Departments of Global Health, Pediatrics, Medicine, and Epidemiology, University of Washington, Seattle, WA, USA.

**Keywords:** antiretroviral, infant, HIV, neutropenia, breastfeeding, PMTCT

## Abstract

Maternal antiretroviral treatment (ART) is recommended for prevention of mother-to-child HIV-1 transmission (PMTCT), including in women with high CD4^+^ cell counts. Within a pediatric HIV-1 vaccine trial PedVacc 002, we assessed hematologic profiles of infants born to mothers receiving ART. All mothers had CD4^+^ cell counts of >350 mm^−3^; 93% received zidovudine-containing ART; infants received nevirapine up to 6 weeks and cotrimoxazole after 6 weeks. Among 84 infants at 19 weeks, 58% had hematologic toxicity; 44% had neutropenia and 23% had anemia. Breastfeeding was associated with 3.8-fold higher risk of neutropenia (RR 3.8, 95% CI 1.03–14.1, p = 0.008). Hematologic monitoring and PMTCT regimen selection are important for optimizing infant outcomes.

## Introduction

Mother-to-child transmission of HIV-1 has been significantly decreased by use of antiretroviral therapy (ART).^1^ However, antiretroviral drugs are concentrated in amniotic fluid and breast milk, raising concerns of toxicity among exposed infants.^2,3^ In utero, fetal exposure to ART among non-breastfeeding infants is associated with hematologic toxicities including anemia, neutropenia, lymphopenia, low CD4/CD8 ratio and thrombocytopenia that may persist up to 15 months of age.^4–6^ Exposure of infants to maternal ART through breast milk is also associated with anemia and neutropenia.^1,7^ Direct infant ingestion of nevirapine (NVP) prophylaxis is also associated with neutropenia.^8^ In addition, the antibiotic cotrimoxazole (trimethoprim/sulfamethoxazole), which is used for prevention of infections among HIV-1-exposed infants, is associated with neutropenia.^9^ With increasing use of early maternal ART, starting at 14 weeks gestation for prevention of mother-to-child HIV-1 transmission (PMTCT), prolonged ART use during breastfeeding, and concomitant use of infant NVP and cotrimoxazole prophylaxis, there is a need to investigate potential hematologic toxicities and their clinical relevance in infants exposed to these drugs.

We conducted a study evaluating hematologic profiles at the age of 19 weeks in a cohort of HIV-1-exposed uninfected infants enrolled in a phase I/II pediatric HIV-1 vaccine clinical trial (PedVacc 002), in which mothers received ART for PMTCT.

## Methods

### Ethics and regulatory approvals.

Approvals for PedVacc 002 were obtained from University of Nairobi/Kenyatta National Hospital Ethics and Research Committee (P266/10/2008) and the Pharmacy and Poisons Board (Ref No. ECCT/08/25/10), Oxford Tropical Medicine Research Ethics Committee (Ref. 52–08), University of Washington Human Subjects Division Internal Review Board (No. 35079) and the Stockholm Regional Ethics Committee (Diary No. 2009/1591–31/1). The study was conducted according to the principles of the Declaration of Helsinki (2008) and complied with the International Conference on Harmonization Good Clinical Practice guidelines.

### Subjects.

Between March and November 2010, we recruited and enrolled 104 HIV-positive pregnant women in Nairobi, Kenya to participate in a randomized clinical trial (RCT) evaluating the safety and immunogenicity of a candidate HIV-1 vaccine MVA.HIVA.^10^ Women were recruited from antenatal clinics at the Kenyatta National Hospital, the largest teaching and referral hospital in Kenya, and from various Nairobi City Council Clinics including Mathare North, Kariobangi, and Kayole, all located in the eastern part of the city. Informed consent was obtained from all women in the study and the infant’s father or another family member co-signed the consent form for participation of the infants. Inclusion criteria for the mother were: 2nd or 3rd trimester of pregnancy, hemoglobin level >9.5 g.dl^−1^, CD4^+^ cell count >350 mm^−3^, neutrophil count >750 mm^−3^ and WHO HIV clinical stage 1 or 2. Participants were provided with ART for PMTCT according to the WHO Option B guidelines, consisting of zidovudine (ZDV) or tenofovir (TDF), lamivudine (3TC), and lopinavir/ritonavir (LPV/RTV) or efavirenz (EFV) or nevirapine (NVP) during pregnancy, delivery and throughout the breastfeeding period. Women selected their preferred feeding option following counseling; formula milk was provided for those who elected replacement feeding. During infant feeding counseling, all mothers were advised to feed their infants on demand with a rough guide of three-hourly feeds in the first few weeks of life. For women who elected replacement feeding, infant formula was provided as needed based on the estimated infant intake per week. Frequency of breast-feeding was assessed by the mother’s self-report when the child was 2, 6, 10, 14, and 19 weeks old. At the age of 19 weeks, most infants were either exclusively on formula or exclusively breastfeeding. Inclusion criteria for infants were: birth weight >2500 g, less than three days old and with no congenital abnormalities. All infants received NVP prophylaxis for the first six weeks of life and received cotrimoxazole prophylaxis from six weeks of life throughout the breastfeeding period. Formula fed infants stopped cotrimoxazole at 10 weeks if the 6-week HIV-1 DNA test was negative. During scheduled study visits, a standard questionnaire on infant health and immunization was completed. At 19 weeks, a HIV-1 test and complete blood count (CBC) were done.

### Hematology.

Hematologic toxicities were defined using the Division of AIDS (DAIDS) table for grading severity of adverse events (version 1.0 December 2004; clarification August 2009). Severity of anemia was categorized by hemoglobin (Hb) level as: Normal (Hb > 11 g.dl^−1^), Grade 1-mild (10–10.9 g.dl^−1^), Grade 2-moderate (9.0–9.9 g.dl^−1^), Grade 3-severe (7.0–8.9 g.dl^−1^) and Grade 4-life threatening (Hb < 7.0 g.dl^−1^). Neutropenia severity was categorized as: Normal (>1300 mm^−3^), Grade 1 (1000–1300 mm^−3^), Grade 2 (750–999 mm^−3^), Grade 3 (500–749 mm^−3^) and Grade 4 (<500 mm^−3^). Leucocyte counts of >2500 mm^−3^ and platelet counts of >125000 mm^−3^ were considered normal. Lymphopenia in children less than 13 years is not graded by the DAIDS table, because the absolute counts are variable; we defined reduced lymphocytes in our infant population (using reference ranges derived from healthy Tanzanian infants) as a lymphocyte count of <3300 mm^−3^.^11^ Any Grade 3 or 4 hematologic toxicity was confirmed by retest.

### Statistical analysis.

We summarized descriptive statistics using medians, interquartile ranges and proportions. We analyzed potential cofactors for hematologic toxicity such as mother’s ART regimen and infant feeding method. Categorical and continuous variables were compared using Fisher exact tests and Wilcoxson rank sum tests, respectively, and risks were compared using relative risks and odds ratios. Data were analyzed using STATA 11 software (Stata Corporation, College Station, Texas, USA).

## Results

### Cohort.

Among 85 HIV-1-exposed infants with hematologic data available at 19 weeks, 1 infant was excluded due to HIV-1 infection. Mothers had a median age of 27 years and were enrolled at median gestation 28.5 weeks; median maternal CD4^+^ cell count was 538 cells/mm^−3^ and hemoglobin 11.5 g.dl^−1^ at enrollment. The majority (60%) of women were already on ART at the first screening visit. Most women (93%) received ZDV-based ART regimens. The median infant birth weight was 3125 g and most (82%) were breastfed. At 19 weeks, the median WHO weight for age Z (WAZ) score was −0.62 (Table 1).

### Infant hematologic profile at 19 weeks.

The median infant neutrophil count was 1350 mm^−3^ and 37 (44%) had neutropenia, with Grade 1 neutropenia in 17 (20%), Grade 2 in 16 (19%), Grade 3 in 3 (4%) and Grade 4 in 1 (1%). The median infant hemoglobin level was 11.6 g.dl^−1^; 19 (22%) were anemic, with Grade 1 anemia in 16 (19%), Grade 2 in 2 (2%) and Grade 3 in 1 (1%). One (1%) of the infants had low lymphocyte count. None of the infants had thrombocytopenia or leucopenia. The median infant white cell count, lymphocyte count and platelet count were 9250 mm^−3^, 6150 mm^−3^ and 424500 mm^−3^, respectively (Table 1).

### Risk factors for hematologic toxicity.

Breastfed infants had a lower mean neutrophil count when compared to formula fed infants (1300 mm^−3^ versus 1800 mm^−3^; p = 0.01). Breast feeders whose mothers were on ZDV-based regimes had lower neutrophil count compared to formula feeders whose mothers were on ZDV-based regimens during pregnancy (1300 mm^−3^ versus 1900 mm^−3^; p = 0.01) (Table 2). The relative risk of neutropenia was almost four times higher in breast fed compared to formula fed infants (RR 3.8, 95% CI (1.03, 14.1), p = 0.008). There was a trend for lower white blood cell counts in breast-feeding infants overall (9100 mm^−3^ versus 9900 mm^−3^, p = .06) and in the subset of ZDV-exposed infants (9100 mm^−3^ in breast-feeders versus 10000 mm^−3^ in formula feeders, p = .08). Categorizing breast feeding mothers by whether the reported number of breastfeeds during the past week was above or below the median, we found a higher relative risk among the more frequently breastfed infants (odds ratio = 5.63 vs. 4.18, p-value for trend .046). There were no significant differences in hemoglobin levels (or anemia), lymphocytes or platelets between breastfed and formula fed infants, overall or in the ZDV-exposed subset.

## Discussion

In this study of infants born to HIV-1-infected mothers receiving combination ART, 44% of infants had neutropenia detected at a single evaluation time-point at approximately 19 weeks of age; 5% had severe to life-threatening neutropenia (Grades 3 or 4). Anemia was common, seen in 22%, but severe anemia was rare (1%). Breastfeeding was associated with a 3.8-fold increased relative risk of any grade of infant neutropenia at 19 weeks compared to formula feeding. Of note, the majority of women were on ART that contained ZDV (a known myelotoxic drug), so that breast milk ingestion of ZDV possibly contributed to the increased neutropenia observed in breastfed infants. The finding of a trend towards a higher risk of neutropenia among the more frequently breast fed infants provides further support for this hypothesis. There is variability in the reported rates of hematologic toxicity among infants exposed to maternal ART. Our finding of a 5% risk of severe neutropenia among infants is similar to that of the MASHI study in Botswana, in which prevalence of severe neutropenia among 69 breastfed infants aged 2–7 months whose mothers received ART was 5.9%.^7^ Unlike the MASHI study, in which women were started on ART at CD4^+^ cell counts below 200 cells/mm^−3^, women in our study initiated ART at higher CD4^+^ cell counts (>350 mm^−3^). Another study from Botswana (Mma Bana) observed higher rates of severe neutropenia (>15%) in six-month old infants of mothers on ART.^12^ In that study, infants received ZDV for four weeks, which could have increased rates of severe neutropenia; however, infants in the MASHI study also received prophylactic ZDV for one month. In the multi-site Kesho Bora study (Kenya, Burkina Faso, South Africa), in which the majority (78%) of infants were breastfed, 8% and 14.6% of infants of mothers on ART had severe neutropenia and anemia (grade 3 or 4), respectively, at birth, and both prevalences decreased to less than 1% by three months of age. Infants in that study received a single dose of NVP at birth and one week of ZDV.^1^

Risk of neutropenia among infants exposed to maternal ART in the MASHI study was increased three-fold compared to infants with no maternal ART exposure.^7^ We found an almost four-fold higher risk of neutropenia among breast feeders compared to formula feeders. This is possibly due to the effects of maternal ART ingested via breast milk. Previous studies have described high levels of ART in breast milk with concentrations of ZDV, 3TC and NVP of 3.21, 3.34 and 0.67 times those in maternal serum four hours after intake of ART respectively.^3^ The finding of a dose response relationship between frequency of breastfeeding and neutropenia has not been explored previously and requires further evaluation. Concurrent cotrimoxazole prophylaxis given to breastfed infants for a longer duration when compared to formula feeders may also have contributed to higher neutropenia rates.^9^ However, a previous study comparing infants in the Mma Bana and MASHI studies who did not receive cotrimoxazole with a cohort that received both cotrimoxazole and ART showed no difference in the risk of severe neutropenia by cotrimoxazole exposure.^13^ In our study, none of the cases of infant neutropenia were symptomatic, a finding consistent with other studies.^1,7^ The clinical significance of neutropenia is still largely unknown, though a study among HIV-1-exposed European infants reported staphylococcal infections related to neutropenia.^4,5,14^

A strength of our study is that it includes evaluation of hematologic toxicities in the current PMTCT context, in which pregnant mothers are started on triple ART with higher CD4^+^ cell counts and at lower WHO stages, and NVP and cotrimoxazole are used for infant prophylaxis. One limitation of the study is that DAIDS criteria may overestimate hematologic abnormalities in Africans^14,15^: Among healthy Zimbabwean infants, mean absolute neutrophil counts were less than half the standard values.^15^ Thus, in the absence of local reference ranges, prevalence of neutropenia may have been overestimated. However, the associations we observed between breastfeeding and neutropenia would remain valid despite regional differences in thresholds for defining severe neutropenia. Another limitation is that we evaluated hematologic parameters only at a single time-point and lacked a control group of HIV-1 unexposed infants at the same sites. Additionally, the study had too few infants to assess the impact of neutropenia on morbidity.

With the current momentum to scale up universal maternal ART in an effort to eliminate pediatric HIV-1, it remains critical to be vigilant regarding infant hematologic toxicities. Use of hematologic ranges and grading systems derived from African infants may be important to avoid overestimation of toxicities. Further studies are necessary to evaluate long-term implications of neutropenia in HIV-1-exposed uninfected infants in resource-limited settings.

## Figures and Tables

**Table 1.** Sociodemographic and laboratory characteristics of mothers and infants.

CHARACTERISTIC n = 84	MEDIAN/NUMBER	IQR/%
**Maternal characteristics during pregnancy**		
Age in years	27	23, 31
Gestational age (weeks)	28.5	24, 32
CD4^+^ cell count (mm^−3^)	538	435, 659
Hemoglobin level (g.dl^−1^)	11.5	10.6, 12.5
Ever taken ART	55	65
Currently on ART	50	60
Containing ZDV	39	78
ZDV-based ART antenatal clinic*	77	93
ZDV-based ART at delivery	78	93
**Infant**		
Birth weight (g)	3125	2900, 3400
WAZ** at 19 weeks	−0.6	−1.3, 0.1
Breast feeders	69	82
**Infant hematologic characteristics at 19 weeks**		
Hemoglobin (g.dl^−1^)	11.6	11, 12.3
Normal Hemoglobin	65	77
Any anemia (DAIDS)	19	23
Grade 1 & 2 (9.0–10.9 g.dl^−1^)	18	21
Grade 3 (7.0–8.9 g.dl^−1^)	1	1
Neutrophils (mm^−3^)	1350	1050, 1970
Normal neutrophil count	47	56
Any neutropenia (DAIDS)	37	44
Grade 1 & 2 (750–1300 mm^−3^)	33	39
Grade 3 & 4 (<500–749 mm^−3^)	4	5
White blood cells (mm^−3^)	9250	7550, 10850
Normal white cell count	84	100
Lymphocytes (mm^−3^)	6150	5200, 7640
Normal (>4000 mm^−3^)	83	99%
Low	1	1%
Platelets (mm^−3^)	424500	326000, 524000
Normal platelets	84	100
Any hematologic toxicity	49	58

* n = 83 due to one missing observation.

** WAZ = Weight for Age Z score.

**Table 2.** Infant hematologic parameters in breastfed and formula fed infants, overall (n = 69, 15 respectively) and in infants exposed and unexposed to ZDV (n = 66, 12).

	BREAST FED (n = 69)	FORMULA FED (n = 15)	RELATIVE RISK	P-VALUE*
Neutrophil count (median)	1,300	1,800		.01
Exposed to ZDV	1,300	1,900		.01
Neutropenia: n (%)	35 (51)	2 (13)	3.8	.008
Hemoglobin (median g.dl^−1^)	11.6	11.1		0.6
Exposed to ZDV	11.6	11.1		0.4
Anemia: n (%)	14 (20)	5 (33)	0.61	0.27
White blood cells (median mrrr^−3^)	9100	9900		.06
Exposed to ZDV	9100	10000		.08
Lymphocytes (median mm^−3^)	6000	6900		.37
Exposed to ZDV	6000	7000		.52
Platelets (mrrr^−3^)	413000	477000		.28
Exposed to ZDV	417000	441000		.72

* Wilcoxon rank sum test for comparison of medians and Fisher exact test for RR.
